# Obstructive Sleep Apnea Syndrome in Heavy Truck Drivers: A Portuguese Prospective Cohort Study

**DOI:** 10.7759/cureus.54047

**Published:** 2024-02-12

**Authors:** Cátia Pimentel, Diana Amorim, Cláudia Santos, Ana Macedo, Salvato Feijó

**Affiliations:** 1 Pulmonology, Centro Hospitalar de Leiria, Leiria, PRT; 2 Clinical Specialist, Escola Superior de Tecnologia da Saúde de Lisboa, Lisbon, PRT

**Keywords:** risk factors for osa, motor vehicle accidents, sleepiness, heavy truck drivers, obstructive sleep apnea

## Abstract

Introduction: Heavy truck drivers with untreated obstructive sleep apnea (OSA) are at higher risk of driving accidents. This study aims to estimate the prevalence of OSA and to identify the most frequent symptoms and comorbidities in heavy truck drivers.

Methods: This cohort study included the employees of a Portuguese transport company between 2019 and 2022. A home sleep apnea test (HSAT) was performed on all patients. SPSS® was used for statistical analysis, and a p-value lower than 0.05 was considered statistically significant.

Results: A total of 86 truck drivers were included, with a mean age of 48.02 years (min. 24, max 66) and a mean body mass index (BMI) of 30.14±4.4 kg/m². After performing an HSAT, it was found that 77.9% of drivers (n=67) had OSA, with a mean apnea-hypopnea index (AHI) of 16.72±14.69 events/hour. Concerning diagnosed patients, 44.78% (n=30) had mild, 31.32% (n=23) moderate, and 20.89% (n=14) severe OSA. Obesity, hypertension, and dyslipidemia had a statistically significant association. There were no statistically significant differences between patients with and without type II diabetes mellitus. The presence of nighttime and daytime symptoms had a statistically significant correlation with OSA diagnosis. Despite only eight patients reporting a high score on the Epworth Sleepiness Scale (ESS), 14 patients reported previous episodes of falling asleep while driving, which might be associated with the non-valorization of daytime sleepiness in these patients. The patients who reported previous episodes of falling asleep while driving were older and had higher BMI, higher ESS, and higher AHI.

Conclusions: In the evaluated truck drivers, the prevalence of OSA was very high (77.9%), which reinforces the importance of screening for this pathology since, when left untreated, it is a major risk factor for exercising their profession safely.

## Introduction

Obstructive sleep apnea (OSA) is characterized by recurrent episodes of partial or complete collapse of the upper airway that obstructs breathing during sleep which often leads to acute derangements in gas exchange, recurrent arousals, activation of the sympathetic nervous system, and disruption in normal hormone secretion, encompassing cardiovascular, neurological, respiratory, and endocrine systems. It is crucial to understand the implications of sleep-disordered breathing, especially due to the significant role that excessive daytime sleepiness (EDS) plays in motor vehicle accidents, pilot errors, and cognitive decline among older adults [1].

Studies reveal that OSA is prevalent. In 2002, the Sleep Heart Health Study found that 24% of men and 9% of women had at least mild OSA. This disease is highly underrecognized, and it is estimated that 82% of men and 93% of women in the United States with OSA are undiagnosed [2]. In Portugal, a cross-sectional study with voluntary notification by Sentinel Doctors revealed that the prevalence of OSA in patients ≥25 years old was 0.89%, which is higher in males (1.47%) and in the age group 65-74 years (2.35%). Obesity (74.2%), arterial hypertension (75.9%), and diabetes mellitus (34.1%) were the most frequent comorbidities; male gender (OR: 2.6) and obesity (OR: 4.0) were associated with a higher risk of severe OSA. A lower prevalence compared with other countries was observed, which may be related to the case definition or reveal an underdiagnosis of this clinical condition [3].

OSA in truck drivers

Professional drivers have high rates of obesity and associated medical conditions. Studies have shown that drivers with multiple comorbidities, including cardiovascular and cerebrovascular diseases, psychiatric conditions, diabetes, medication usage, uncorrected visual defects, and obesity, are at a heightened risk of being involved in motor vehicle crashes [4]. A meta-analysis that included 26 studies, involving a total of 6794 commercial drivers who were suspected of having OSA, concluded that body mass index (BMI) and history of hypertension were significant test elements in the more accurate prediction models [5].

Driving a motor vehicle is a multifaceted complex task involving sensory perception, sound judgment, adequate response time, and appropriate physical capability. OSA may impair these driving prerequisites and adversely affect driving ability, possibly resulting in a crash causing death or injury [6]. It is essential to acknowledge that EDS poses a significant hazard on the road, potentially compromising the driver's alertness and reaction time [6].

Tools such as the Epworth Sleepiness Scale (ESS) can be used as subjective measures of EDS. However, these tests rely on honest reporting by the driver, and there is evidence that incorrect reporting may occur in some cases. The tools are therefore just one aspect of the comprehensive assessment. A history of frequent self-reported sleepiness while driving or motor vehicle crashes caused by sleepiness also indicates a high risk of motor vehicle crashes [6].

Fatigue and drowsiness contribute to approximately 15-20% of accidents on monotonous roads, particularly highways. Collisions resulting from sleepiness often involve veering off the road or rear-ending another vehicle. Insufficient sleep often stemming from prolonged periods of wakefulness, chronic sleep deprivation caused by extended work hours, shift work, or various medical and neurological conditions can cause EDS [7].

OSA is the predominant medical condition associated with EDS, making it a significant risk factor for both drowsy driving and car accidents caused by falling asleep at the wheel [7]. Additional research has revealed a significant increase in the rate of motor vehicle crashes, ranging from two to seven times higher, among individuals with sleep apnea compared to control subjects. Studies have also demonstrated increased objectively measured sleepiness while driving (electroencephalography and eye closure measurements) and impaired driving-simulator performance in people with confirmed sleep apnea. The level of impairment observed is comparable to that caused by illegal alcohol intoxication or sleep deprivation. Drivers with severe sleep-disordered breathing may have a higher rate of crashes than those with a less severe sleep disorder [6].

Prompt diagnostic assessment, effective treatment, and comprehensive education for patients and their families are essential measures to reduce the occurrence of sleepiness-related crashes among high-risk drivers with OSA. By addressing OSA and its associated symptoms, the prevalence of accidents caused by sleepiness can be mitigated.

Objectives

As OSA is an underdiagnosed disease with a high impact on driving safety, this study aims to estimate the prevalence of OSA and to identify the comorbidities and the most prevalent symptoms associated with OSA among heavy truck drivers.

A preliminary analysis of the data presented in this article was previously presented as a meeting abstract at the European Respiratory Society (ERS)/European Sleep Research Society (ESRS) Sleep and Breathing Conference 2023 on April 20, 2023.

## Materials and methods

A cohort study was carried out at Centro Hospitalar de Leiria-Hospital Santo André in Leiria, Portugal, that included truck drivers of a Portuguese national and international transport company between 2019 and 2022. The researchers provided detailed information regarding OSA, the potential implications of the diagnosis and the implications of daytime sleepiness while driving, and all necessary procedures and requirements for study enrollment to the drivers. All participation was voluntary and anonymized in accordance with the Helsinki Declaration.

Approval for the said study was obtained from Comissão de Ética da Unidade Local de Saúde da Região de Leiria, EPE (approval number: 89/CECHL/2023). Included were all the truck drivers who were interested in enrolling in this study. In contrast, excluded were truck drivers not signing the informed consent and/or with a previous diagnosis of OSA.

The drivers were asked to fill out medical questionnaires on daytime sleepiness, symptoms of OSA, sociodemographic data, and comorbidities associated with OSA. Daytime sleepiness was measured by the ESS. Screening was carried out with a home sleep apnea test (HSAT) using the Alice PDx® (Philips, Amsterdam, Netherlands) and ApneaLink Air® (ResMed, San Diego, California, United States) equipment, previously validated for OSA screening. Trained study personnel instructed the participants on how to connect all the equipment.

Data were downloaded and analyzed using the default settings for episodes of apnea, hypopnea, and desaturation. A respiratory event resulting in a complete lack of airflow as measured by a reduction greater than 90% in the thermal sensor for 10 or more seconds was classified as apnea and a reduction in nasal pressure of at least 30% for 10 seconds or longer with 3% or greater oxygen desaturation was classified as hypopnea. The current diagnostic criteria for OSA established in 2014 by the American Academy of Sleep Medicine were used, and all drivers diagnosed with OSA were referred to a Pulmonology consultation for further evaluation. SPSS® was used for statistical analysis, and a p-value lower than 0.05 was considered statistically significant.

## Results

Eighty-six truck drivers were enrolled in this study, all male, with a mean age of 48.02±9.99 years (min. 24, max 66) and a mean BMI of 30.14±4.4 kg/m². After performing HSAT, it was found that 77.9% of drivers (n=67) had OSA, with a mean apnea-hypopnea index (AHI) of 16.72±14.69 events/hour. Concerning the diagnosed patients, 34.88% (n=30) had mild, 26.74% (n=23) moderate, and 16.28% (n=14) severe OSA, as detailed in Table 1.

**Table 1:** Patient distribution according to OSA severity OSA: obstructive sleep apnea; BMI: body mass index

	Non-OSA	Mild OSA	Moderate OSA	Severe OSA
n (%)	19 (22.09%)	30 (34.88%)	23 (26.74%)	14 (16.28%)
Age (in years)	40.05±10.24	48.30±9.42	52.87±7.59	50.29±8.46
BMI	28.01±5.17	29.30±3.76	31.14±3.66	33.17±4.22

In this sample, obesity had a statistically significant association with a higher AHI (p=0.001), as specified in Table 2. The mean AHI of patients with normal weight was 6.11±7.51/h (n=9), overweight 12.65±9.37/h (n=38), obesity class 1 23.33±17.24/h (n=24), and obesity class 2 21.16±17.49/h (n=14), and only one patient had obesity class 3 (AHI 44/h).

**Table 2:** Mean AHI according to comorbidities AHI: apnea-hypopnea index

Comorbidities		n (%)	Mean AHI	p-value
Hypertension	Yes	24 (27.91%)	22.23±17.39/h	p=0.016
	No	62 (72.09%)	14.59±13.04/h	
Type II diabetes	Yes	9 (10.47%)	10.70±6.31/h	p=0.642
	No	77 (89.53%)	15.53±14.72/h	
Dyslipidemia	Yes	22 (25.58%)	21.27±15.65/h	p=0.032
	No	64 (74.42%)	15.16±14.14/h	

A statistically significant difference was also evident between patients with arterial hypertension and a higher AHI (p=0.016), which was 14.59±13.04/h in patients without hypertension and 22.23±17.39/h in patients with arterial hypertension (n=24), as detailed in Table 2. There were no statistically significant differences between patients with (n=9) and without type II diabetes mellitus. In this sample, patients with dyslipidemia (n=22) had a higher AHI (21.27±15.65/h) than patients without dyslipidemia (15.16±14.14/h), with a statistically significant difference (p=0.032).

The most frequent symptoms in this population are described in Table 3.

**Table 3:** Symptoms associated with OSA OSA: obstructive sleep apnea

Symptoms		n (%)	p-value
Nighttime	Snoring	71 (82.56%)	0.251
	Nocturia	27 (31.4%)	0.184
	Vivid dreams	26 (30.23%)	0.886
	Witnessed apneas	23 (26.74%)	0.088
	Gasping	14 (16.28%)	0.526
Daytime	Fatigue	35 (40.7%)	0.151
	Xerostomia	33 (38.37%)	0.493
	Difficulty concentrating	28 (32.56%)	0.228
	Falling asleep while driving	14 (16.28%)	0.444
	Morning headaches	12 (13.95%)	0.314
	Epworth >9	8 (9.3%)	0.025

Despite the presence of any nighttime symptoms (p=0.034) or daytime symptoms (p=0.008) correlated with higher AHI, only an Epworth >9 had an isolated direct correlation with OSA diagnosis (p=0.025). In this population, the mean Epworth was 4.30±3.2/h. The ESS was statistically different between patients with AHI <5/h (2.89±1.97) and AHI ≥5/h (4.70±3.37), with a p-value of 0.025. There was also a difference between patients with AHI <15/h (3.39±2.52) and AHI ≥15/h (5.51±3.61), with a p-value of 0.002. The Pearson correlation between the AHI and ESS was 0.479, indicating a positive correlation between these two scores. Despite only eight patients reporting a high Epworth score, 14 patients reported previous episodes of falling asleep while driving. The patients who reported previous episodes of falling asleep while driving were statistically significantly older and had higher BMI, higher ESS score, and higher AHI as detailed in Table 4.

**Table 4:** Characteristics of the patients who reported falling asleep while driving BMI: body mass index; ESS: Epworth Sleepiness Scale; AHI: apnea-hypopnea index

	Falling asleep while driving		p-value
	No	Yes	
Age	46.99±9.87	53.36±9.12	0.029
BMI	29.59±4.23	32.95±4.61	0.007
ESS	3.79±2.74	6.93±4.12	0.004
AHI	14.75±12.57	26.88±20.38	0.021

All patients who had an OSA diagnosis were referred to a Sleep Medicine consultation. Auto-titrating continuous positive airway pressure (auto-CPAP) was prescribed in 36 patients (10 patients with mild OSA, 14 with moderate OSA, and 12 with severe OSA). Ten patients were newly diagnosed and were awaiting evaluation in a Pulmonology consultation. Four patients refused ventilation and are under evaluation for other therapeutic options.

## Discussion

Heavy truck drivers in safety-sensitive roles encounter specific difficulties when it comes to accessing the diagnosis and treatment of OSA. One of the primary challenges is the potential lack of awareness or some anxiety regarding the impact of this diagnosis on their employment. These factors can result in the underreporting of symptoms, ultimately leading to underreferral and underdiagnosis. Encouraging anonymity has in fact proven to be effective in enhancing the reporting of symptoms and addressing this issue [8].

Accurate symptom reporting by all transportation operators is imperative, as clinical decision-making relies heavily on the data provided to healthcare personnel. Moreover, factors such as inadequate sleep, irregular work schedules, and circadian misalignment can significantly amplify safety hazards. It is crucial to note that healthcare providers are unable to assess these risks in real-time. Consequently, the onus lies on the individuals employed in safety-sensitive positions to proactively educate themselves, identify potential risks, and refrain from operating if they have concerns about impairment. This responsibility entails working in close cooperation with their healthcare team [8].

As supported by our study, the presence of nighttime symptoms and daytime symptoms correlated with higher AHI. Despite that, all clinicians should be aware that when evaluating a transportation operator, the absence of symptoms does not imply the absence of risk. In one study of commercial drivers whose prevalence of OSA was 77.7%, results showed that 47.1% were objectively sleepy based on multiple sleep latency testing, but none reported sleepiness when asked. Therefore, self-reported assessments of sleepiness may not correlate with laboratory-based assessments [8]. As seen in this population, despite only eight patients having an Epworth score above 9 points, 14 patients disclosed previous episodes of falling asleep while driving.

The latest position paper of the American Academy of Sleep Medicine states that workers who perform safety-sensitive functions in the transportation industry should be screened for OSA using both self-reported symptoms and established, objectively measurable criteria such as blood pressure and body weight [9]. A comprehensive understanding of OSA and its associated effects on multiple physiological systems is essential for addressing its potential consequences and implementing appropriate interventions.

In the United States, employers are advised to implement OSA management programs even in the absence of a regulatory requirement. Currently, examiners should refer to the 2016 Medical Review Board (MRB) recommendations as a starting point for identifying at-risk drivers who should be referred for diagnostic testing for suspected OSA. These recommendations involve established criteria that are risk factors for OSA, such as BMI, micrognathia or retrognathia, airway and neck size, age, gender, and history of comorbid hypertension, type II diabetes, stroke, coronary artery disease, or arrhythmias. The recommendations also involve self-reported symptoms such as fatigue or sleepiness during the wake period, loud snoring, and witnessed apneas. Similar criteria were developed in 2006 by a joint task force of physicians and scientists [9,10].

Our population had an AHI statistically significantly higher in patients with obesity, hypertension, and dyslipidemia, and we were able to demonstrate that there is a positive correlation between these comorbidities and OSA diagnosis. Therefore, it is crucial to address the specific healthcare needs of professional drivers and implement targeted interventions to mitigate the increased crash risk associated with OSA and their underlying medical conditions.

OSA has a significant impact on sleep quality, daytime sleepiness, psychomotor vigilance, and driving performance in simulated environments. As a result, untreated OSA can lead to a two- to three-fold increase in the risk of crashes compared to individuals without OSA. The primary symptom associated with increased crash risk and fatal accidents in patients with OSA is subjective daytime sleepiness. However, even patients without daytime sleepiness can experience an elevated crash risk. Nevertheless, effective treatment of OSA seems to normalize the risk of accidents. Therefore, identifying professional drivers with OSA and providing them with effective treatment can significantly reduce crash-related injuries and fatalities while improving drivers' overall health [4].

Treating OSA with CPAP has demonstrated positive outcomes in reducing daytime sleepiness and minimizing the risk of crashes among individuals with OSA. CPAP has also been shown to enhance driving-simulator performance to the same levels as the control group. This suggests that CPAP therapy not only reduces the risk of drowsy driving and drowsy driving crashes but also improves driving abilities, ensuring safer and more reliable performance behind the wheel [7].

Nonetheless, our study does have the limitation of using an HSAT to define OSA. Although portable monitors have been proposed as a cheaper and more accessible technology for OSA diagnosis in specific populations, such as individuals with high pretest suspicion and without complicated comorbidities, the gold standard test remains in-laboratory, technician-monitored polysomnography (PSG).

## Conclusions

In the evaluated truck drivers, OSA prevalence was very high (77.9%), which reinforces the importance of screening for this pathology. Most importantly, OSA is a treatable disease, and successful treatment can reduce crash risk to levels similar to those observed in individuals without OSA. In the ongoing management of OSA among truck drivers, it is essential to incorporate education, continuous monitoring of treatment efficacy, and interventions to promote adherence to treatment protocols. These measures play a crucial role in ensuring OSA optimal management and enhancing safety in the transportation industry.
